# The ER quality control and ER associated degradation machineries are vital for viral pathogenesis

**DOI:** 10.3389/fpls.2014.00066

**Published:** 2014-03-11

**Authors:** Jeanmarie Verchot

**Affiliations:** Department of Entomology and Plant Pathology, Oklahoma State UniversityStillwater, OK, USA

**Keywords:** plant virus interactome, virus–host interactions, virus–membrane interactions, unfolded protein response, ubiquitin proteasome system, chaperones, ERAD

## Abstract

The endoplasmic reticulum (ER) is central to protein production and membrane lipid synthesis. The unfolded protein response (UPR) supports cellular metabolism by ensuring protein quality control in the ER. Most positive strand RNA viruses cause extensive remodeling of membranes and require active membrane synthesis to promote infection. How viruses interact with the cellular machinery controlling membrane metabolism is largely unknown. Furthermore, there is mounting data pointing to the importance of the UPR and ER associated degradation (ERAD) machineries in viral pathogenesis in eukaryotes emerging topic. For many viruses, the UPR is an early event that is essential for persistent infection and benefits virus replication. In addition, many viruses are reported to commandeer ER resident chaperones to contribute to virus replication and intercellular movement. In particular, calreticulin, the ubiquitin machinery, and the 26S proteasome are most commonly identified components of the UPR and ERAD machinery that also regulate virus infection. In addition, researchers have noted a link between UPR and autophagy. It is well accepted that positive strand RNA viruses use autophagic membranes as scaffolds to support replication and assembly. However this topic has yet to be explored using plant viruses. The goal of research on this topic is to uncover how viruses interact with this ER-related machinery and to use this information for designing novel strategies to boost immune responses to virus infection.

## INTRODUCTION

The endoplasmic reticulum (ER) and Golgi apparatus comprise a fundamental endomembrane compartment for de novo protein synthesis. In the last 20 years, researchers have begun to uncover the protein quality control (QC) machineries that are housed in the ER and that tightly regulate protein production (Brandizzi et al., 2003; Zhao and Ackerman, 2006; Urade, 2009; Moreno and Orellana, 2011; Parmar and Schroder, 2012; Verchot, 2012). The ER QC machinery provides: (a) chaperone-assisted protein folding and assembly; (b) post-translational modification of proteins; and (c) protein transport out of the ER for maturation and secretion. The ER is also the major site for synthesis of membrane related phospholipids and membrane embedded proteins (Fagone and Jackowski, 2009). Cellular membranes are essential to compartmentalize functions, manage energy production, storage, and cell-to-cell communication. Cellular membranes function to segregate environments for protein synthesis, modification, secretion, and degradation.

Proteins that do not successfully progress through these mechanisms are categorized as malformed proteins and are subjected to ER associated degradation (ERAD). Typically, the toxic accumulation of malformed proteins as the result of biotic or abiotic stress, activates the unfolded protein response (UPR), which is a signaling network initiated at the ER. Protein sensors that reside in the ER (such as IRE1 and PERK) respond to ER stress by increasing transcription of a set of genes encoding ER resident chaperones (Parmar and Schroder, 2012) to enhance the protein folding capacity of the ER. Malformed proteins that cannot be refolded are sequestered, modified by ubiquitination, and degraded by the 26S proteasome (Meusser et al., 2005; Muller et al., 2005). The ubiquitin proteasome system (UPS) is a major regulatory system that contributes to all aspects of cell biology, not just ERAD. Both the ER QC and UPS machinery are widely conserved among eukaryotes. Researchers are just beginning to understand the role of the UPS in plant virus infection and immunity.

Serving as a model process in the field of stress biology, positive strand RNA viruses infecting mammals and plants pose an enormous biosynthetic burden on the ER. To aid cellular adaptation to infection, viruses trigger vigorous membrane and protein synthesis, and/or protein transfer to the Golgi apparatus (Netherton et al., 2007). Host gene expression is transiently enhanced to adapt to the immediate needs of virus gene expression, mitigate ER stress, and create a cellular environment that tolerates virus infection. Invading viral pathogens manipulate the ER QC machinery to: (1) support replication and protein production; (2) accommodate the translational needs of defense related transcripts; (3) subvert components of the system in a manner that promotes infection (Jelitto-Van Dooren et al., 1999). Researchers are working to determine when viruses highjack the intact ER QC machinery to promote viral protein production and when viruses isolate critical ER chaperones and divert them for other processes that are essential to infection (Nagy et al., 2011; Verchot, 2012). One notion is researchers have considered is that membrane expansion to accommodate virus infection is somehow linked to UPR induction early in infection. However, recent data with *dengue virus* (DEN) indicates that early induction of UPR is correlated with membrane rearrangement and synthesis but is not directly responsible for changes in membrane composition (Pena and Harris, 2012). Thus, it is not clear if or how these two early events are directly linked.

In mammalian systems, researchers have described the relationship between the UPR, autophagy and oxidative stress (Ke and Chen, 2011b). Viruses that cause a huge burden to global health such *as hepatitis C virus* (HCV) and DEN exploit the UPR to modulate autophagy and oxidative stress pathways. This is essential to promote virus replication and evade host immunity (Ciccaglione et al., 2007; Urade, 2009; Costa et al., 2010; Jouan et al., 2012; Shinohara et al., 2013). The mechanism by which these and other viruses subvert the UPR is not resolved (Diehl et al., 2011; Estrabaud et al., 2011). In plants, UPR and autophagy are linked to plant immune responses (Moreno et al., 2012). The gene expression profiles of *potato virus X* (PVX) infected hosts have revealed enhanced transcription of ER QC machinery needed to expand the protein folding capacity of the ER (Ye and Verchot, 2011; Ye et al., 2013). Nonetheless, there have been fewer studies in plants examining the role of UPR and autophagy in immune evasion by plant viruses.

## PLANT VIRAL INTERACTIONS WITH CELLULAR MEMBRANES ARE ESSENTIAL FOR REPLICATION AND EGRESS

In support of virus replication, positive strand RNA viruses cause extensive reorganization of cellular membranes and create subcellular compartments, called “viroplasms” (Castellano and Martelli, 1984; den Boon et al., 2010; Verchot, 2011). Such membrane bound compartments provide a protective environment and maintain the necessary viral and host proteins in proximity to the genomic template. Plant viruses belonging to the genera *bromovirus*, *comovirus*, *dianthovirus, nepovirus, pecluvirus*, *potexvirus*, recruit ER membranes to create viroplasms (Carette et al., 2000; Dunoyer et al., 2002; Ritzenthaler et al., 2002; Turner et al., 2004; Diaz et al., 2010; Verchot, 2011) while *potyviruses* use both ER and chloroplast membranes (Wei and Wang, 2008; Wei et al., 2010). Furthermore, many viruses induce massive membrane synthesis or alter the lipid composition of certain membranes needed for formation of these replication centers. In particular, *brome mosaic virus* (BMV), *cowpea mosaic virus* (CPMV), *grapevine fanleaf mosaic virus* (GFLV), and are among the well-studied examples (Carette et al., 2000; Ritzenthaler et al., 2002; Noueiry and Ahlquist, 2003). However, the signal transduction mechanism that controls ER membrane proliferation and their phospholipid profiles has not yet been elucidated.

Many plant RNA viruses encode movement proteins that interact with an active ER network to move from cell to cell across plasmodesmata (Boevink and Oparka, 2005; Tilsner et al., 2010, 2011; Genoves et al., 2011). Most known plant virus movement proteins are categorized into one of four recognized superfamilies (Melcher, 2000). The 30K superfamily encodes movement proteins related to the *tobacco mosaic virus* (TMV) 30K movement protein. Viruses belonging to the 30K superfamily are reported to encode movement proteins that carry viral genomic RNAs across the plasmodesmata. The TMV 30K protein, in particular, transports replication complexes across plasmodesmata in a manner that is dependent on both the ER and microfilament networks (Kawakami et al., 2004; Wright et al., 2007; Guenoune-Gelbart et al., 2008; Sambade et al., 2008; Niehl et al., 2013; Zavaliev et al., 2013). Two other key superfamilies are viruses that encode small hydrophobic movement proteins that insert into the ER. The double gene block (DGB) superfamily include viruses belonging to the genera *carmovirus, closterovirus*, *panicovirus*, and *sobemovirus*; and the triple gene block (TGB) superfamily includes viruses belonging to the genera *allexivirus, benyvirus, carlavirus, foveavirus, hordeivirus, pecluvirus*, and *potexvirus* (Vilar et al., 2002; Peremyslov et al., 2004; Sauri et al., 2005; Martinez-Gil et al., 2007, 2010; Verchot-Lubicz et al., 2010). In addition, the enveloped *tospovirus, tomato spotted wilt virus* encodes glycoproteins that localize to ER-export sites and Golgi complexes (Ribeiro et al., 2008).

## EUKARYOTIC VIRUSES INTERACT WITH THE UPR MACHINERY TO PROMOTE PATHOGENESIS

As mentioned previously, positive strand RNA plant viruses pose an enormous biosynthetic burden on the ER, creating a higher than normal protein load. Therefore virus infection increases the potential for malformed proteins to accumulate thereby contributing to ER stress (Noueiry and Ahlquist, 2003). In this regard, the re-establishment of ER homeostasis by upregulating the ER protein folding and degradation machineries appears to be a coordinated adaptive response to virus invasion (Jelitto-Van Dooren et al., 1999; Urade, 2009; Liu et al., 2011; Wahyu Indra Duwi et al., 2013).

The best studied examples linking UPR to RNA virus infection are members of the family *flaviviridae*, such as DEN-2, HCV, *japanese encephalitis virus* (JEV), and *west nile virus* (WNV; Yu et al., 2006; Ambrose and Mackenzie, 2011; Paradkar et al., 2011). Flaviviruses depend upon the ER/Golgi network for replication and mature virions are released by budding through membranes of the ER/Golgi network. Three proteins prM, E, and NS1 enter the secretory system and are modified by glycosylation. Other non-structural proteins NS2A, NS2B, NS4A, and NS4B remain anchored to the ER. Each *flavivirus* has its own signature for activating the IRE1/XBP1-signaling pathways with unique benefits to virus infection (Iwata and Koizumi, 2012). For example, XPB1 activates genes involved in protein folding, ER biogenesis, and the ER degradation enhancing a-mannosidase-like protein 1 (EDEM-1; Mai and Breeden, 1997; Nekrutenko and He, 2006; Acosta-Alvear et al., 2007; Li et al., 2009). WNV NS4A and NS4B, as well as the HCV NS4B proteins activate XBP1 without altering EDEM-1 transcription (Zheng et al., 2005; Ambrose and Mackenzie, 2011). Researchers speculate that both HCV and WNV manipulate XBP1 signaling to promote the production of ER resident chaperones and membrane proliferation needed to support virus replication and protein production (Zheng et al., 2005; Ambrose and Mackenzie, 2011). The HCV E1 and E2 proteins are also reported to activate IRE1/XBP1 signaling events as well as PERK related oxidative stress pathways (Chan and Egan, 2005). For HCV, activation of these other pathways is linked to suppressing innate immunity while promoting virus replication (Ke and Chen, 2011a). The NS1 glycoprotein of JEV and DEN-2, as well as the NS2B/NS3 polyprotein of DEN-2 activate the XBP1-signaling pathway. Silencing XBP1 does not interfere with DEN-2 or JEV infection, but does exacerbate the cytopathic effects of these viruses. This suggests that the UPR is manipulated by these viruses to promote infection and counter host innate immunity (Estrabaud et al., 2011; Pena and Harris, 2011).

There are a few recent examples of plant viruses which interact with components of the UPR machinery to promote infection (**Table 1**). In plants IRE1 splices the bZIP60 transcription factor mRNA as a first step in UPR signaling (Deng et al., 2011; Iwata and Koizumi, 2012; Moreno et al., 2012). bZIP60, like XBP1, is reported to upregulate expression of the ER chaperone network that provides QC (Wahyu Indra Duwi et al., 2013) and likely benefits plant RNA virus infection. Importantly, it is not known whether the bZIP60 signaling pathway or other signaling pathways is responsible for the induced expression of membrane biosynthetic genes or changes in the host protein degradation patterns needed for virus infection. One virus example is PVX, which is a *potexvirus*. The PVX TGB3 movement protein is an 8 kDa movement protein that is tethered to the ER, induces expression of bZIP60 and ER resident chaperones as BiP, protein disulfide isomerase (PDI), and calreticulin (CRT; Garcia-Marcos et al., 2009; Ye and Verchot, 2011). Silencing bZIP60 gene expression in protoplasts greatly inhibited PVX replication. These data argue that although TGB3 is a movement protein, it nevertheless contributes to the regulation of virus replication by its impact on host gene expression (Ye and Verchot, 2011). Preliminary experiments indicated that BiP plays a role in preventing cytotoxic cell death during PVX infection which suggests that the UPR is an early event essential for persistent PVX infection and benefits virus replication (Jelitto-Van Dooren et al., 1999; Xu et al., 2005; Slepak et al., 2007; Urade, 2007). Two other virus examples include *papaya ringspot virus* (PRSV) and TMV, which require CRT to promote virus movement and possibly block calcium-dependent host defenses (**Table 1**). Since CRT is also regulated by bZIP60, it is worth further investigation to learn if PRSV interacts with this UPR signaling pathway. It is noteworthy that in our experiments, TMV did not appear to induce bZIP60 expression in a manner that is similar to PVX, which suggests that TMV could usurp CRT for its own processes without manipulating bZIP60 expression (Ye and Verchot, 2011; Ye et al., 2013). It is not known if silencing bZIP60 alters TMV or PRSV infection and such experiments are needed to better understand the role of UPR sensors in these virus infections.

**Table 1:** Membrane related host proteins or post-transcriptional regulatory networks for compatible plant-virus interactions.

Virus genus	Virus species^a^	Cellular protein or machinery^b^	Type of association	Viral protein partner	Post-transcript regulatory network	References
*potexvirus*	PVX	bZIP60 SKP1	mRNA induction	TGB3	UPR and UPS turnover of TGB2	Ye and Verchot (2011), Ye et al. (2013)
*tobamovirus*	TMV	CRT	Direct protein interaction	30K	Plasmodesmata and virus movement	Chen et al. (2005)
	ToMV	UPS	Verified protein ubiquitination	30K	Protein modification and turnover, regulates virus movement.	Reichel and Beachy (2000)
*nepovirus*	GFLV	CRT	Co-localization	Movement protein	Receptor for viral movement protein at cell surface or in cytosol	Laporte et al. (2003)
*potyvirus*	PRSV	CRT	Direct protein interactions	HC-Pro	Interfere with plant Ca^+2^ signaling to block host defenses	Shen et al. (2010), Sahana et al. (2012)
	PRSV, LMV, PVY,	PPAA,alpha1 subunit of 2proteasome	Direct protein interactions	HC-Pro	Regulates protein turnover, HC-pro interaction	Ballut et al. (2005), Jin et al. (2007), Sahana et al. (2012)
	PSbMV	Poly ubiquitin	mRNA expression	Un-known	Unknown role in virus infection	Aranda et al. (1996)
*polerovirus*	BWYV	SCF E3 ubiquitin ligase	Potential interaction	P0 protein	P0 is an F box protein that inserts into the SCF complex and targets AGO1 for degradation	Pazhouhandeh et al. (2006), Baumberger et al. (2007), Bortolamiol et al. (2007)
*enamovirus*	PEMV	SCF E3 ubiquitin ligase	Potential Interaction	P0 protein	P0 is an F box protein that inserts into the SCF complex and targets AGO1 for degradation	Fusaro et al. (2012)
*tombusvirus*	TBSV	Nedd4-type Rsp5p ubiquitin ligase	Direct protein interactions	P92 replicase	Turnover of p92 replication protein	Barajas et al. (2009)
	TBSV	Cdc34p ubiquitin conjugating enzyme	Direct protein interactions	P33 replicase	Modify P33 necessary for interaction with host ESCRT1	Li et al. (2008), Barajas and Nagy (2010)
*tymovirus*	TYMV	UPS pathway	Protein interactions	66K replicase 69K move-ment protein	Modify and turnover of viral RdRp and movement protein	Hericourt et al. (2000), Drugeon and Jupin (2002), Camborde et al. (2010)

a Virus names: PVX, potato virus X; TMV, tobacco mosaic virus; toMV, tomato mosaic virus; GFLV, grapevine fanleaf virus; PRSV, papaya ringspot virus; LMV, lettuc mosaic virus; PVY, potato virus Y; PSbMV, pea seedborne mosaic virus; BWYV, beet western yellow virus; PEMV, pea enation mosaic virus; TBSV, tomato bushy stunt virus, TYMV, turnip yellow mosaic virus.

b In this column UPS is identified when it is known that the virus protein is known to be ubiquitin modified and turned over. Protein interactions are verified by the ubiquitin modification, but the relationship with the proteasome is typically evidence by turnover not by direct protein interaction assays. However, there are times when the exact ubiquitin related enzyme is known and identified in this column. These are verified by either yeast two hybrid or affinity chromatography.

In general, viruses have evolved to exploit the UPR machinery as a means to create environments that are favorable to infection. The UPR and ERAD mechanisms, by which plants and mammals respond to ER stress, have some significant similarities. While there is a greater body of research describing a role for XBP1-signaling pathways in mammalian virus infection, new evidence linking bZIP60 signaling pathways to plant virus infection suggest that RNA viruses infecting eukaryotes may generally manipulate the UPR to cope with ER stress, promote virus infection while reducing cytopathic effects, and possibly alter antiviral immunity.

There are also reports that viruses can perturb the cross talk between UPR signaling and other stress pathways including oxidative stress, autophagy, type I IFN antiviral response, and innate immune responses (Tardif et al., 2005; Sir et al., 2008; Garcia-Marcos et al., 2009; Ambrose and Mackenzie, 2011; Estrabaud et al., 2011; Evans et al., 2011; Yue et al., 2012). The exact mechanisms by which plant and mammalian RNA viruses manipulate UPR is not yet known, but given the universality of UPR regulation, the same machinery is likely to play an equally important role in plant virus replication and should be studied in more depth.

### THE CONTRASTING ROLES OF CALRETICULIN IN STRESS RESPONSE AND VIRAL PATHOGENESIS

The expression of ER resident chaperones is transcriptionally coordinated in response to ER stress and bZIP60 is one of the identified transcription factors responsible for increased expression of a network of ER resident folding enzymes and chaperones. Among these are CRT and calnexin (CNX), which are highly conserved proteins critical to processing nascent glycoproteins and calcium homeostasis in the ER. In *Arabidopsis* there are three CRT isoforms (CRT1a, CRT1b, and CRT3) while in mammals there are only two CRT isoforms. CRT1a and CRT1b are similar isoforms that play general roles in maintaining protein folding and calcium levels in the ER (Persson et al., 2003; Thelin et al., 2011). The transcription of AtCRT1a and AtCRT1b is often coordinated, and is much more highly induced than AtCRT3 by such ER stress inducing compounds as tunicamycin (Jia et al., 2009; Christensen et al., 2010). Overproduction of CRT in response to pathogen attack or stress would increase the Ca^2^^+^ buffering capacity of the cell. Thus viruses could potentially target CRT gene expression to create an environment that is favorable to virus infection.

In contrast to mammalian systems, plant CRTs localize to several subcellular compartments. AtCRT1a/b also associate with plasmodesmata and research suggests that it plays a role in Ca^2^^+^ homeostasis in the plasmodesmata (Baluska et al., 1999; Christensen et al., 2010; Thelin et al., 2011). Localization of CRT to plasmodesmata requires the N-terminal signaling sequence for ER insertion, which implies that CRT moves through an ER-dependent route to reach the plasmodesmata (Wyatt et al., 2002). The AtCRT1a/1b co-localizes with the TMV movement protein in plasmodesmata (Chen et al., 2005; Ye et al., 2013). CRT1a was shown to bind TMV movement protein *in vitro* and *in vivo* by affinity chromatography, yeast two hybrid analysis, and fluorescence resonance energy transfer microscopy. Overexpression of CRT hinders TMV cell-to-cell movement and blocks movement protein accumulation in the plasmodesmata (Chen et al., 2005). GFLV also interacts with CRT to promote cell-to-cell movement. GFLV uses a tubule guided movement mechanism for intercellular movement (Laporte et al., 2003). Researchers proposed that CRT binds the viral movement protein and serves as a base for tubular assembly. The viral encoded movement protein moves through the secretory system to the destination where they form oligomers that build into tubules. Tubules extend across plasmodesmata and carry virion particles between neighboring cells (Laporte et al., 2003). Given that TMV and GFLV are not known to impact CRT expression, these data provide further support to the notion that TMV and GFLV are more likely to subvert CRT1a/1b from their normal function to promote plant virus infection through interactions with the viral movement proteins.

All three CRT isoforms contain nuclear targeting signals in their central domain and C-terminal HDEL ER-retention signal. The wheat TaCRT3 was reported to translocate to the cytoplasm and nucleus (Jia et al., 2008; An et al., 2011). In *Nicotiana *ssp., CRT also localizes to the Golgi and plasma membrane (Jia et al., 2008; Matsukawa et al., 2013). Investigations of transcriptionally co-regulated gene networks in plants suggest that CRT1a and CRT1b are co-expressed with many ER chaperones while CRT3 is co-expressed with pathogen related signal transduction genes (An et al., 2011; Thelin et al., 2011). Given that transcriptionally coordinated genes typically provide related functions, the function of CRT3 is likely diverged from these ER resident isoforms.

As mentioned previously, PRSV and PVX represent another class of viruses that interacts with CRTs at the level of gene expression. Researchers showed that papaya CRT1a/b interacts with the PRSV HC-Pro protein using yeast two hybrid and BiFC assays in plant cells (Shen et al., 2010). HC-Pro is a well-studied viral protein that is involved in multiple functions including virus movement and suppression of post-transcriptional gene silencing (Shiboleth et al., 2007; Yap et al., 2009). As mentioned earlier, the PVX TGB3 movement protein increases the expression of bZIP60 and several ER resident chaperones including CRTs in *Arabidopsis* and *Nicotiana benthamiana* leaves. Silencing bZIP60 in protoplasts hampers PVX infection which led us to reflect on whether upregulation of ER chaperones, such as CRT1b is a necessary result of TGB3 activation of bZIP60. Considering that PRSV, like PVX, induces expression of CRT, it is reasonable to speculate that PRSV might interact with the co-expression gene network to enlist the ER QC machinery. However, the relationship of bZIP60 to a co-expression network involving CRT is not yet known. Further research is needed to better understand the roles of CRT in PRSV and PVX infection and determine if there are separate types of interactions involving plant viruses and such components of the ER QC machinery. Knowledge of whether bZIP60 is capable of activating CRT gene expression is also important for understanding how viruses interact with bZIP60 related gene networks.

**FIGURE 1:**
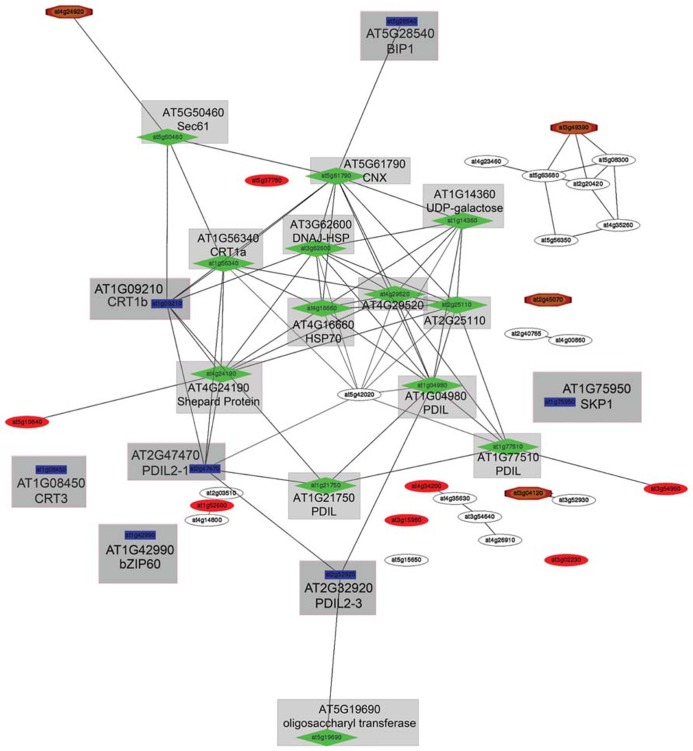
**Visualization of the genes associated with UPR in plants as determined by GeneCAT analysis.** This is a simple subnetwork example (Mutwil et al., 2008). The nodes represent genes and the lines represent the relationships between the genes. The genes identified in blue are the query genes. The nearest co-expressed genes are highlighted in green and form a cluster of co-expressed genes based on an analysis of mutual co-expression ranks between the top 50 genes from the list. The bold lines, in this subnetwork, identify those genes that have low mutual ranks according to GeneCAT webtool analysis (Mutwil et al., 2008; Usadel et al., 2009). Query genes including bZIP60, SKP1, and CRT3 do have lines connecting them to the co-expressed gene network arguing that they are not connected in a manner that is statistically relevant. Thus the evidence that these genes are upregulated early in virus infection could suggest that the virus may be interacting with more than one signaling pathway. Each blue and green labeled node, has a gray box with the name of the gene and its accession highlighted in larger print.

To better understand the relationship of bZIP60 with ER QC machinery, we took advantage of the GeneCat co-expression analysis webtool (Mutwil et al., 2008; Usadel et al., 2009) to examine the whether the *Arabidopsis* bZIP60 (AT1G42990), CRT1b (AT1G09210), and CRT3 (AT1G08450) provide a chaperone framework for ER QC (**Figure 1**). Evidence that these genes are transcriptionally coordinated (using an *r*-cutoff value < 0.7) when induced during biotic stress, would support the hypothesis that they are related functionally and contribute to similar processes. Evidence of a weak relationship would suggest that bZIP60 expression is not solely tied to the expression of these genes. We also included SKP1 (AT1G75950) in the analysis query because it is induced alongside bZIP60 and CRT by PVX infection and is a co-factor in the UPS machinery that does not reside in the ER. This analysis identified genes that show strong rankings (when no average *r*-cutoff value was applied), appear to form a cluster that is connected within one or two nodes to the query genes, and appear to be mutually co-expressed. The GeneCat analysis output revealed that the expression of CRT1b, BIP1, PDI-like protein 2-1 and 2-3 (PDIL2-1, PDIL2-3), which are all components of the ER QC machinery, is strongly coordinated (**Figure 1**). The relatedness of these genes was also reported by Thelin et al. (2011) using the webtool PlaNet to explore the co-expression networks involving CRT. The comparative analysis using the query genes CRT1b, CRT3, and bZIP60, also demonstrates that CRT1b is co-expressed with ER chaperones and folding enzymes while CRT3 is either weakly linked or not linked to this network. Given reports linking CRT3 to a subnetwork of genes involved in pathogen-related signaling events, these data confirm that these CRTs have divergent functions (Thelin et al., 2011). Interestingly, there is no evidence that expression of bZIP60 is transcriptionally coordinated with the ER QC machinery when we use strict or relaxed cutoff (*r* < 50) to generate the network. CRT3 and SKP1 also lie outside the network and their expression is not coordinated with bZIP60 expression (**Figure 1**). It is possible that co-expression analysis does not reveal the regulatory relationships linking bZIP60 and CRTs and that these genes are linked in other ways. For example there might be other intermediate factors that connect to bZIP60 these ER resident chaperones.

## THE PRO-VIRAL ROLE OF UPR AND AUTOPHAGY

Chaperone mediated autophagy is extensively studied in yeast and mammals and is recently identified to play an important role in infection and immunity in plants. In mammals, accumulation of malformed proteins in the ER activates UPR via PERK, IRE1, and ATF6 and all three sensors are required for induction of autophagy. Autophagy is a well-known degradation pathway for organelles and cytoplasmic components. Initially, the cellular autophagy protein LC3 associates with membranes forming crescent-shaped double membrane structures that sequester misfolded proteins and damaged organelles. These structures mature to form double membrane vesicles known as mature autophagosomes. These autophagosomes can fuse with endosomes to form amphisomes which then become acidified due to the presence of vacuolar ATPases and then fuse with the lysosomes to form autolysosomes that degrade their cargo.

*Polivovirus* (PV), HCV, DEN, and JEV are a few examples of the many positive strand RNA viruses that induce autophagic signaling and subvert autophagosomes or amphisomes to use as scaffolds supporting replication and assembly (Taylor and Jackson, 2009; Shi and Luo, 2012; Richards and Jackson, 2013). Since autophagy is also a pathway to degradation, viruses have developed strategies to block lysome fusion. Shi and Luo (2012) describe autophagic flux as the balance between the rate autophagosome formation and degradation. This concept is interesting with respect to virus infection, because viruses can act at different steps in the process to increase autophagosome formation, alter the rate of amphisome conversion, and reduce degradation, thereby triggering an incomplete autophagic response. DEN, for example localizes with immature autophagosomes. DEN and JEV are both enveloped viruses that rely on receptor-mediated endocytosis for cellular uptake. One possibility is that autophagosome-endosome fusion is a factor in virus entry and uncoating. HCV relies on autophagosome formation to support virion assembly and autolysosome for suppressing host immune responses. PV uses autophagosomes as a scaffold for virus replication and the acidic amphisomes to promote virus assembly (Richards and Jackson, 2012, 2013).

With regard to plant viruses, there is no information yet that indicates a pro-viral role for autophagy in the infection process. Researchers have linked autophagy to plant innate immunity involving TMV infection. N-gene mediated immune response to TMV includes a form of programmed cell death known as a hypersensitive response (HR). In this regard, autophagy limits the extent of cell death to a local area on a leaf, preventing uncontrolled spread of HR throughout healthy tissues. This serves to contain the immune response to a localized region (Liu et al., 2005; Li et al., 2012).

## MANIPULATING THE UBIQUITIN PROTEASOME SYSTEM FOR VIRUS INFECTION

The ubiquitin-26S proteasome system is the prevailing route for protein removal and is widely conserved among eukaryotes (Smalle and Vierstra, 2004; Vierstra, 2009). The ubiquitin conjugating pathway depends on the host E1, E2, and E3 ligases to link ubiquitin moieties to a protein substrate (Miura and Hasegawa, 2010). Certain types of ubiquitin modifications predestine a protein for degradation by the proteasome while others determine alternative subcellular locations or activations. Thus the ubiquitin ligase machinery, which includes the F-box protein that harnesses the substrate, modifies intact as well as malformed proteins. Ubiquitination can also be reversed by the action of de-ubiquitinating enzymes (DUBs) which can either trim a polyubiquitin chain or remove it from the substrate (Chenon et al., 2012).

There is a growing body of evidence that plant and mammalian viruses interact with both ubiquitin ligases and DUBs. There are many cases where eukaryotic viruses manipulate the UPS machinery to avoid immune clearance. For example the *human immunodeficiency virus* (HIV) Vpu protein is a low molecular mass protein with a single transmembrane domains that inserts into the ER. Vpu binds to the cellular CD4 protein in the ER and recruits the human F-box protein bTrCP targeting CD4 for degradation via the ubiquitin-proteasome pathway. CD4 is a cell surface receptor required for HIV uptake into cells, and the process of dislocation and degradation of CD4 in the ER reduces the number of available receptors at the cell surface and is important to free HIV gp160 in the ER for virus maturation and trafficking (Bour et al., 1995; Belaidouni et al., 2007; Malim and Emerman, 2008; Nomaguchi et al., 2008).

Among plant viruses, the UPS machinery can be manipulated to degrade components of the cell’s gene silencing machinery, thereby promoting virus infection. *Poleroviruses* and *enamoviruses* encode the P0 protein which contains an F box protein motif (Baumberger et al., 2007; Fusaro et al., 2012). It is reported that the P0 inserts into the SCF complex and enables degradation of ARGONAUTE1 (AGO1) which is a core component of the RISC complexes and is an essential component of the RNA-silencing machinery. P0 acts as a silencing suppressor that enables AGO1 degradation, compromising RNA silencing, as well as the degradation of targeted viral RNAs. Furthermore, interactions between the *beet western yellow virus* (BWYV) P0 protein and SKP1 modulates programmed cell death during virus infection. Mutations that interrupt the ability of P0 to interact with SKP1 result in systemic necrosis, suggesting that the P0–SKP1 complex P0 is a silencing suppressor protein which might target components of the silencing machinery for proteasomal degradation through its interactions with SKP1 (Pazhouhandeh et al., 2006).

PVX is also known to interact with SKP1 but the role of this cofactor in virus infection is not yet clarified (Ye et al., 2013). As mentioned earlier the PVX TGB3 movement protein associates with the ER and upregulates expression of the bZIP60 transcription factor and several ER resident chaperones early in virus infection. TGB3 also induces SKP1 expression alongside several other genes suggesting that both the UPR and UPS systems are upregulated to handle the increased protein load in the cell. Another possibility is that TGB3 enhances the capacity of the UPS to degrade key host proteins that are either related to or independent of the RISC complex. The PVX TGB1 protein acts as a silencing suppressor protein that targets AGO1 for proteasomal degradation (Bayne et al., 2005; Chiu et al., 2010). It is worth to consider that TGB3 acts in concert with TGB1 to promote the degradation of AGO1.

Replication of *turnip yellow mosaic* *virus *(TYMV; a *tymovirus*) is broadly affected by the UPS and is a prime model for comparison with other positive strand RNA viruses. The TYMV genome encodes two non-structural proteins of 69 kDa and 206 kDa in size. The viral coat protein is expressed from a subgenomic RNA. The TYMV 69K protein is a viral movement protein that can be polyubiquitinated. Reports indicate that the 69K protein is turned over by the proteasome (Drugeon and Jupin, 2002). Protein turnover is suggested to either regulate virus movement or reduce cytotoxic accumulation of viral proteins. There are other viral movement proteins that are also regulated by proteasomal turnover including the TMV 30K movement protein and coat protein, the *polerovirus* 17 kDa movement protein, and the PV TGB3 (Mas and Beachy, 1999; Jockusch and Wiegand, 2003; Pazhouhandeh et al., 2006; Ju et al., 2008). These combined reports suggest that plant viruses may generally target the UPS to regulate the stability of virus movement proteins.

The TYMV 206 kDa polyprotein contains domains which provide methyltransferase, proteinase, helicase, and RNA-dependent RNA polymerase (RdRp) activities (Martelli et al., 2007). The proteinase domain autocatalytically cleaves the 206 kDa polyprotein to generate a 66K RdRp and a 140K protein which are both present in the active replicase complex. The 66K RdRp is modified by ubiquitin during infection and is a target for UPS turnover (Hericourt et al., 2000). Thus the UPS regulates TYMV replication (Camborde et al., 2010). The 140K protein is a precursor product that is further cleaved to produce the mature 98K proteinase and 42K helicase. The proteinase domain within the 140K or 98K protein also possesses deubiquitylating (DUB) enzyme activity (Chenon et al., 2012). Research suggests that 66K RdRp is the substrate for the deubiquitylating activity of these proteins. TYMV replication occurs along chloroplast membranes and the 66K protein is transported to this location by the driving interactions with the 140K protein. It is reported that the 140K or the 98K proteins monitor the ubiquitin moieties attached to the 66K RdRp to stabilize the replicase complex and promote virus replication early in infection. Then later in virus infection, ubiquitin modification of the 66K leads to proteasomal degradation and shuts down replication. Thus, DUB activity also plays a role in directing viral RNA replication (Chenon et al., 2012).

*Tomato bushy stunt virus *(TBSV) is a member of the genus *tombusvirus* and provides another example whose replicase is impacted by the UPS machinery. Ubiquitination of the TBSV p33 controls interactions with the host ESCRT protein which is important for subcellular targeting of the viral replicase. The UPS system also monitors the accumulation of the TBSV p92 protein which is essential for virus replication (Li et al., 2008; Barajas et al., 2009; Barajas and Nagy, 2010). These combined examples suggest that there might be a common viral strategy to manipulate or alter the ubiquitin-mediated degradation machinery to promote plant virus infection.

## CONCLUSION

Positive strand RNA viruses depend heavily on the ER for genome synthesis, protein production, and cell-to-cell movement. Given the biosynthetic burden that viruses pose on the cell, sustaining ER homeostasis and creating a membrane rich environment is an essential adaptation for infection to succeed. One question that remains to be answered is whether the extensive membrane expansion and remodeling is a means to compensate for the translational burden on the ER caused by virus infection, or is the direct outcome of UPR and/or autophagy. Given that virus translation causes a burden on the ER QC machinery, it is not known for certain whether this is the proximal cause of ER stress and activation of the UPR. It is arguable that the impact of translation on the ER causes cells to adapt and survive and that UPR activation is a component of cellular adaptation. However, there is a growing body of evidence to that indicates UPR is not just a cell survival response in the face of a toxic infection, but that many positive strand RNA viruses act in a targeted manner to upregulate UPR and benefits critical steps in virus infection. The examples provided here suggest that positive strand RNA viruses encode effector proteins that activate specific branches of the UPR in a targeted manner. For example, the HCV E1 and E2 trigger the IRE1/XBP1 pathway; the WSN NS4A also triggers the XBP1 pathway but does not activate EDEM-1; the JEV and DEN-2 NS1 proteins also activate XBP1 pathway; and in plants the PVX TGB3 activates the bZIP60 pathway.

One of the benefits of UPR activation is the increased availability of cellular chaperones which typically drive substrate protein folding and complex assembly in various cellular compartments. It seems obvious that viruses require the cell to have a greater protein folding capacity to accommodate the translational burden caused by infection, but viruses have an additional need to subvert certain chaperones from their normal function to help drive events during the infection process. As summarized in **Table 1**, we presented examples of *potyviruses, nepoviruses*, and *tobamoviruses* that pirate CRT to promote intercellular transport. However, the literature does not show whether many of these viruses activate signaling mechanisms to stimulate CRT expression. Furthermore, it is not known if other components of the ER QC machinery are diverted from their cellular roles to viral protein complexes. In general, virologists are currently working to uncover the parameters that determine which ER resident chaperones engage with viral proteins to promote viral pathogenesis and whether this benefits infection at the expense of cellular homeostasis or host immunity.

The relationship of the UPR to membrane biosynthesis or re-organization is not established but it is reasonable to predict that researchers are likely to be able to explain the mechanisms behind virus induced membrane synthesis as we explore the need for autophagic membranes. For example, membrane synthesis might be stimulated, not directly by the UPR machinery but by viruses interactions with the autophagic machinery. Perhaps viruses act on a parallel pathway to stimulate membrane synthesis needed for autophagosome and amphisome production. Given that viruses require changes in gene expression relating to both membrane synthesis and cellular chaperones, it would be intriguing to learn how the signal transduction events that relate the UPR and autophagy are connected.

The role of ubiquitin and DUBs in virus infection is an intriguing new topic with great potential to provide new insights into the host machinery involved in regulating virus infection. We presented examples of viruses belonging to a broad number of virus genera interact with the UPS machinery to either regulate its own replication cycle, modulate intercellular movement, or evade host defenses. As an aggregate, this work shows that viruses can manipulate the UPS machinery to suppress host defenses, modulate virus replication, and regulate viral protein turnover. The breadth of examples clearly shows that the UPS machinery plays a critical role in virus infection for a wide range of plant viruses.

## Conflict of Interest Statement

The author declares that the research was conducted in the absence of any commercial or financial relationships that could be construed as a potential conflict of interest.
